# “It is better to die”: experiences of traditional health practitioners within the HIV treatment as prevention trial communities in rural South Africa (ANRS 12249 TasP trial)

**DOI:** 10.1080/09540121.2016.1181296

**Published:** 2016

**Authors:** Mosa Moshabela, Thembelihle Zuma, Joanna Orne-Gliemann, Collins Iwuji, Joseph Larmarange, Nuala McGrath

**Affiliations:** a Africa Centre for Population Health, University of KwaZulu-Natal, Mtubatuba, South Africa; b School of Nursing and Public Health, University of KwaZulu-Natal, Durban, South Africa; c INSERM U1219 – Centre Inserm Bordeaux Population Health, Université de Bordeaux, Bordeaux, France; d Université de Bordeaux, ISPED, Centre INSERM U1219-Bordeaux Population Health, Bordeaux, France; e Research Department of Infection and Population Health, University College London, London, UK; f Centre Population & Développement (Ceped UMR 196 UPD IRD), Institut de Recherche pour le Développement, Paris, France; g Faculty of Medicine and Faculty of Social, Human and Mathematical Sciences, University of Southampton, Southampton, UK

**Keywords:** HIV testing, antiretroviral treatment, treatment-as-prevention, traditional healers, HIV stigma, South Africa

## Abstract

The ANRS 12249 Treatment-as-Prevention (TasP) cluster-randomized trial in rural South Africa uses a “test and treat” approach. Home-based testing services and antiretroviral treatment initiation satellite clinics were implemented in every cluster as part of the trial. A social science research agenda was nested within TasP with the aim of understanding the social, economic and contextual factors that affect individuals, households, communities and health systems with respect to TasP. Considering the rural nature of the trial setting, we sought to understand community perceptions and experiences of the TasP Trial interventions as seen through the eyes of traditional health practitioners (THPs). A qualitative study design was adopted using four repeat focus group discussions conducted with nine THPs, combined with community walks and photo-voice techniques, over a period of 18 months. A descriptive, interpretive and explanatory approach to analysis was adopted. Findings indicate that THPs engaged with the home-based testing services and HIV clinics established for TasP. Specifically, home-based testing services were perceived as relatively successful in increasing access to HIV testing. A major gap observed by THPs was linkage to HIV clinics. Most of their clients, and some of the THPs themselves, found it difficult to use HIV clinics due to fear of labelling, stigma and discrimination, and the ensuing personal implications of unsolicited disclosure. On the one hand, a growing number of patients diagnosed with HIV have found sanctuary with THPs as alternatives to clinics. On the other hand, THPs in turn have been struggling to channel patients suspected of HIV into clinics through referrals. Therefore, acceptability of the TasP test and treat approach by THPs is a major boost to the intervention, but further success can be achieved through strengthened ties with communities to combat stigma and effectively link patients into HIV care, including partnerships with THPs themselves.

## Introduction

The ANRS 12249 Treatment-as-Prevention (TasP) Trial in rural KwaZulu-Natal, South Africa is evaluating a universal “test and treat” strategy for the prevention of HIV transmission (Iwuji et al., 2013; Orne-Gliemann et al., 2015). Home-based HIV counselling and testing is offered repeatedly to all members within the defined geographic area of trial communities. In the intervention arm, antiretroviral treatment (ART) is offered to all HIV positive individuals through designated TasP trial clinics regardless of their CD4 count, whilst in the control arm, treatment is offered according to South African national ART guidelines. Whilst the primary outcome of the TasP Trial is to reduce HIV incidence at population level, secondary outcomes include community perceptions and experiences of the universal test and treat approach (Iwuji et al., 2013; Orne-Gliemann et al., 2015). TasP remains a relatively new innovation as a public health intervention at population level, and yet offers much promise for the control of the HIV epidemic. Therefore, TasP is a challenge not only for scientists, policy-makers and healthcare providers, but also for individuals, families and local communities (Orne-Gliemann et al., 2015).

Indeed TasP, through its test and treat approach, is expected to cause major shifts and changes in the way communities perceive and experience the surveillance, monitoring and control of the HIV epidemic (Orne-Gliemann et al., 2015). Since TasP is implemented as a community-level intervention, a better understanding of community-level factors likely to influence the test and treat approach will be fundamental to the social science research agenda. A recent critical review by Underwood, Hendrickson, Van Lith, Kunda, and Mallalieu (2014) indicated that social support networks, cultural and gender norms, and stigma and discrimination were the most important community-level factors associated with the HIV treatment cascade (Underwood et al., 2014). Currently, we know little about such community-level social factors that may influence the success or failure of the universal test and treat approach within TasP. Perspectives of local community members, including traditional health practitioners (THPs), will be fundamental in shaping our understandings of social factors affecting individuals, families and communities. In this study, we report on the local THPs perceptions and experiences of the TasP test and treat approach, a subset of the ANRS 12249 TasP Trial social science research agenda already reported by Orne-Gliemann et al. (2015).

THPs are legally recognised through the South African Traditional Health practitioners Act of 2007, and include diviners, herbalists, traditional surgeons and traditional birth attendants (Peltzer, 2009). Although spiritual and faith healers, often associated with church, religious and prophetic forms of healing, were not included in this Act, they are arguably as prevalent and important as other forms of THPs (Gqaleni, Moodley, Kruger, Ntuli, & McLeod, 2007). However, the World Health Organisation (WHO) does recognise religious healing, and defines a THP as a “a person who is recognised by the community where he or she lives as someone competent to provide health care by using plant, animal and mineral substances and other methods based on social, cultural and religious practices” (WHO, 1978, p. 9). In South Africa, THPs are considered influential figures of authority in South Africa, and initiatives are currently underway to formally recognise them as health service providers by the Department of Health. However, regulatory councils for THPs and their traditional medicines have yet to be established (van Niekerk, 2012). The use of THPs by people living with HIV has been reported to delay access to HIV testing and treatment in rural areas (Audet et al., 2014; Moshabela, Pronyk, Williams, Schneider, & Lurie, 2011). A recent community-level survey in South Africa, at a study site adjacent to the ANRS 12249 Trial, demonstrated that 20% of people taking ART also use traditional healers (Pantelic et al., 2015). Appelbaum-Belisle et al. (2015) found that people living with HIV use ART and THPs for different reasons, indicating a complementary form of utilisation behaviour. As a result, engaging THPs through HIV education and referrals is an approach recommended as a possible solution in helping to link people living with HIV to care, as was demonstrated in Mozambique (Audet et al., 2013). However, there still exist negative and conflicted attitudes between biomedical health workers and THPs. Mutual understanding and trust will need to be achieved for effective collaboration to ensue (Van Rooyen, Pretorius, Tembani, & Ten Ham, 2015). According to a review by Hanson, Zembe, and Ekström (2015), it will become increasingly important to actively engage communities, and THPs as community members, in the control of the HIV epidemic, and begin to promote community-driven interventions (Hanson et al., 2015).

## Methods

### Study setting

Hlabisa health sub-district is one of the five sub-districts in the rural district of Umkhanyakude in northern KwaZulu-Natal, South Africa. Approximately 77% of the sub-district is classified as rural, and 92% of the 228,000 inhabitants speak Zulu as a first language (Solarsh, Benzler, Hosegood, Tanser, & Vanneste, 2002). The rural population lives in scattered homesteads. HIV prevalence in 2011 in the Hlabisa sub-district was 29% among adults (Zaidi, Grapsa, Tanser, Newell, & Bärnighausen, 2013). Healthcare services are provided through 1 central community hospital and 17 fixed primary healthcare clinics. Hlabisa hosts the Wellcome Trust-funded Africa Centre for Population Health. The Africa Centre carries out socio-demographic, HIV and TB surveillance in a geographically defined area covering about 40% of the population in the sub-district (Tanser et al., 2008). The Africa Centre also hosts the ANRS 12249 TasP Trial, which is implemented outside of the Africa Centre demographic surveillance area. The trial area contains 8 of the 17 fixed primary healthcare clinics in the Hlabisa sub-district.

### Study population

Four THPs were purposively sampled for maximum variation in socio-demographic factors through the community engagement unit of the Africa Centre and ANRS 12249 TasP Trial Group. A snowballing technique was used to identify other THPs through home visits. THPs were included if 16 years or older, resident in one of the four Tasp Trial clusters, able to communicate in isiZulu, able to provide informed consent, willing to participate actively in discussions, willing to have discussions recorded, willing to attend repeat interviews, willing to provide their contact details and able to commit time required for the research activities. A total of nine THPs were included, seven females (all above 40 years of age) and two males (both below 35 years of age). In South Africa, most THPs are female and diviners/faith healers, whereas males function more as herbalists.

### Data collection

The first phase of the TasP trial started in March 2012, and ended in March 2014. Focus group discussions (FGDs) were conducted with THPs, so as to explore their collective and consensus perceptions and experiences as a body of practitioners, and repeated four times over a period of 18 months between 2013 and 2014. All THPs attended the first and second meetings, with one dropout in meeting three and two dropouts in the fourth meeting. Venues for the FGDs, allowing privacy, were identified by a local community leader. FGDs were conducted in isiZulu, audio-recorded with consent from participants, and field notes were captured. The duration of FGDs ranged from 60 to 120 minutes. Data were collected on healthcare services and their utilisation within study communities, understanding of TasP approach, local practices to support HIV testing and early ART initiation, and lastly, barriers and facilitators to HIV testing, early ART initiation and adherence. FGDs were transcribed and translated from isiZulu to English. Repeat group discussions were supplemented with observations through community walks (Chambers, 1994) and the Photo-voice technique (Wang & Burris, 1997) on barriers and facilitators.

### Data analysis

Thematic analysis was used to generate themes with emphasis on descriptive, interpretive and explanatory analyses. Data sources were combined and coded for experiences and perceptions relevant to the testing, linkage, treatment, adherence and retention components of TasP. Two researchers (TZ and MM) coded the data using open coding within data sources, and compared codes between data sources using axial coding and coders through peer-auditing procedures. Relations between codes were compared and categories were generated. Data sources were revisited and explored further to identify contradictory statements and new codes, followed by revision of codes, categories and themes. Observation and visual data were used as supplementary sources to produce textual data, and also used in generating supportive and contradictory codes and revision of categories.

### Ethical considerations

The Biomedical Research Ethics Committee (BREC) of the University of KwaZulu-Natal approved the trial (BCF104/11) and the social science programme (BE090/12). The trial was approved by the Department of Health in KwaZulu Natal, and the Medicines Control Council of South Africa. Participation was voluntary, and all participants granted both verbal and written informed consent. Participants could withdraw at any point during the study, and their confidentiality was ensured. All recordings were stored in a password-protected electronic file.

## Results

THPs participating in the study constituted diviners, faith healers and herbalists. Diviners and herbalists provided healing through ancestral messengers, whereas faith healers were often active church members and leaders who provided spiritual healing through prayer and God-sent messengers.

### Home testing opportunity

The availability of home testing for HIV was reported as a critical component of the TasP “Test and Treat” Campaign, and THPs encouraged each other to make use of the service themselves, as well as for their families, neighbours and clients.

One day, two “TasP” fieldworkers went past my home and a colleague of mine told me how helpful they are in testing and giving you results. I then jumped on the opportunity and asked them to test me… They did the HIV test and I was negative. (THP, P7, Diviner, Female, 61)

THPs also identified and reported missed opportunities for HIV testing in the homes, mostly among young people. However, given their power of influence in households and communities, they were able to mobilise people during rounds of home testing, approximately every three months.

The problem that I have is that the kids at home don’t want to check and they even hide when they see people coming to do tests. I need to go after them and encourage them to test otherwise they don’t want to…. (THP, P6, Diviner, Female, 60)

### Finding sanctuary in healing

When HIV was diagnosed, many clients to THPs saw the diagnosis as a form of punishment, and would thus consult THPs in search of reasons for God or their ancestors to subject them to such suffering. THPs who practised as prophets and faith healers reported that their clients sought solace for their diagnosis in the comfort of the church or other forms of spiritual healing. In addition, there is an expectation that church-linked healers should not publically reveal confessions or secrets they know of their members.

…they join the church so they can hide among church members because the rule is that church secrets are not supposed to be spoken outside the church…. (THP, P1, Faith Healer, Female, 52)

Furthermore, clients who are aware of their HIV status also go to THPs claiming that witchcraft is the cause of their illness. As a result, clients would expect THPs to consult with the spirits in search of answers, and they confess their HIV diagnosis to THPs to seek their counsel, further explained through the photo.

When someone is ill from this (HIV) they run to us (traditional healers)… we have a very big challenge. They come to us and say that Makhosi I dreamt at night that someone was giving me idliso (“bewitching” by food)… [While] he knows that the illness is eating him away. (THP, P5, Diviner, Female, 60)

### Failure in linkage to care

THPs further played an active role in linking their clients to care services, by using referral cards designed by the Department of Health. However, the success of this approach was limited, and many of the clients they referred did not present at health facilities, for which THPs expressed frustration.

My patients take the card but throw it away when they leave my home. I always see the cards lying around in the veld, and see that they did not go to the clinic like I had asked them to [group laughs]. (THP, P5, Diviner, Female, 60)

When linkage to care was achieved, some of the participants reported negative experiences with health workers in the HIV clinics, who at times passed judgement based on their knowledge of participants’ life circumstances.

I will make myself as an example when I got sick and went to the clinic, I asked to check my HIV status and they said “where will you be getting HIV since your husband passed away some time ago?”…. (THP, P7, Diviner, Female, 61)

These negative experiences contributed to the discomfort felt by some of the THPs in using clinics, and their failure to return to clinics for follow-up. Since THPs were also members of the community, they expressed shared lived experiences with their clients. THPs also reported examples of clients who avoided their local clinics, largely due to fear of being recognised by other community members.

…some people even change clinics. They don’t use the local clinic [A] that is closer to their homes. They prefer going to town to use [Clinic B]. Others go to [Clinic C], they are from [Village A]. They do not want to be seen by other people who are from the same community. (THP, P9, Diviner, Female, 53)

### Fear of stigmatisation

Participants in this study highlighted the fear of experiencing shameful feelings generally associated with the unintended revelation of HIV status by using HIV-specific clinic facilities, wherein THPs or their clients may be recognised by other community members. The fear of being “the talk” of the community was so intense that even some of the THPs themselves did not want to be associated with the HIV diagnosis. Regardless of such fears, THPs did recognise the importance of seeking health care and receiving treatment.

People in general, including myself, once we know that we are infected, it is difficult to take a decision to go to the clinic because we think it will cause an embarrassment but the clinic is actually very important. (THP, P7, Diviner, Female, 61)

There was a further complication for THPs themselves, in that they are seen as “powerful” and in some ways, “invincible”, and thus succumbing to HIV themselves reduces the perception of “divine power” associated with THPs. For those who refuse either to test for HIV or to reveal their diagnosis, they feel “it is better to die” than to test and treat HIV. Put differently, these THPs adopted the view that it is “better to die with dignity, than live with shame”, even though the perception of dignity was based on non-disclosure.

What I mean to say is that even us (THPs), we don’t want to be known when we become HIV positive because of the stigma attached to being a traditional healer and the expectation that we cannot contract this virus, whereas in actual fact this virus does not discriminate, whether you are a traditional healer, a priest or whoever you are…. (THP, P7, Diviner, Female, 61)

THPs also recognised that other figures of “divinity”, as well as health workers, have also been infected with HIV. THPs participating in this study felt that there was no need for THPs to feel shame when infected with HIV, and those who received HIV education resolved that education was necessary to help steer those misinformed THPs away from misconceptions, such as [THP, P2, Herbalist, Female, 49], whose views were openly challenged by the group members.

Clients who found sanctuary in the church and among THPs included those who refused to test, disclose or link to care due to fear of stigma and discrimination. The key to overcoming this barrier was reported as not simply provision of HIV information, but effective partnerships with HIV organisations to change attitudes and render services accessible to the community.

## Discussion

The experiences of THPs suggest that the HIV home testing component of the ANRS 12249 TasP has been largely successful, and its implementation has influenced community perceptions of HIV testing, as informed by THPs. However, gaps in the linkage to care component were reported by THPs, which will inadvertently bear implications for the ART initiation component of the universal test-and-treat approach. According to the THPs, fear of stigma and discrimination, through unsolicited disclosure to fellow community members by merely attending HIV clinics, as well as the stigmatising practices of health workers, was the most important community-level driver of poor linkage to care. THPs, including both traditional and faith healers, were consulted by community members vulnerable to stigma as providers of sanctuary and solace away from the experiences of discrimination in the community. The need for sanctuary will persists for as long as stigma and discrimination continue to persist. THPs were willing to form partnerships with HIV treatment organisations, including those providing TasP, in order to strengthen linkage to care for those in need of testing as well as those known to be living with HIV and in need of treatment. Furthermore, participants did not address the TasP trial components of early ART initiation, adherence or retention in any direct terms. Further research will be necessary to investigate THPs’ understandings of the specific notion of early treatment initiation, a core component in TasP.

Efforts to combat stigma and discrimination likely form a good basis to forge stronger ties between THPs and the TasP intervention, in line with the proposal by Hanson et al. (2015) to involve communities . Recently, Vermund (2014) eloquently argued that with the advent of TasP, never has the need for stigma reduction been more urgent. In addition to concerns regarding stigma and discrimination, Young, Flowers, & McDaid, 2014 demonstrated that poor HIV literacy can be a barrier to the acceptability of TasP (Young et al., 2014). In this study, THPs who were considered misinformed about HIV often refused to test, disclose or treat HIV, a pattern likely to represent views of their clients. HIV knowledge varies in surveys of THPs, ranging from 38% (Walwyn & Maitshotlo, 2010) to 60% (George, Chitindingu, & Gow, 2013). In their survey of THPs treating HIV patients, George et al. (2013) showed better study outcomes among HIV-trained THPs than their untrained counterparts.

According to Campbell, Skovdal, and Gibbs (2011), there remains a need to create social spaces to provide education and tackle HIV-related stigma, including in churches. While some churches may hold contradictory views to some interventions for HIV, such as condoms, other church spaces hold promise in challenging stigmatising ideas and practices. The same can be said about homes and spaces of THPs in general. This approach is further supported by the literature on social capital and HIV (Campbell, Williams, & Gilgen, 2002), whereby THPs and the social spaces, including churches, can be accessed to build social support networks (Reis, Galvao, & Gir, 2013). These networks have been identified as one of three main determinants of success in HIV care, besides transport and stigma (Underwood et al., 2014). However, the focus of these social spaces needs to remain the fight against HIV stigma, which is a social process contingent on social context, and therefore necessitates contextually relevant interventions to create acceptance of people living with HIV, as well as acceptability of practices of HIV testing and ART (Earnshaw & Chaudoir, 2009; Florom-Smith & De Santis, 2012; Sengupta, Banks, Jonas, Miles, & Smith, 2011).

In conclusion, acceptability of the TasP test-and-treat approach by THPs could be a major boost to the intervention, and further success can be achieved through strengthened ties with communities, including THPs themselves. Further research is necessary to assess the feasibility of THP partnerships within the TasP research agenda, as well as within broader HIV stigma reduction interventions. These partnerships could include HIV education for THPs, and empowering them as HIV educators. THPs could also be offered materials and education to test their clients for HIV, refer those who are diagnosed with HIV, and act as advocates for linkage to care and early ART initiation. Better designed evaluation studies that adopt suitable HIV stigma frameworks to the context of the TasP intervention will be needed, as well as participatory action research approaches involving community members and THPs, including traditional birth attendants who exists on a large scale in other parts of sub-Saharan Africa. THPs can play a more exemplary role in their communities by openly testing for HIV and encouraging others to test, including their fellow THPs, family members and clients. In light of the findings of this study, it remains clear that THPs are well positioned to strengthen both HIV testing as well as linkage to care for their clients. THPs can help create social spaces within religious and traditional health practices for the purposes of combatting HIV-related stigma and discrimination. Furthermore, THPs could act as bridges in building social support networks in the community, and potentially use these social networks to strengthen community participation in the efforts to eliminate HIV within local communities.

## Supplementary Materials

Appendix
