# Supplementary material for: “It is better to die”: experiences of traditional health practitioners within the HIV treatment as prevention trial communities in rural South Africa (ANRS 12249 TasP trial)
Source: AIDS Care. Author manuscript; Available in PMC 2016 Nov 4. (PMC5096678; doi:10.1080/09540121.2016.1181296)
Supplement: Appendix [file NIHMS70179-supplement-Appendix.pdf]

Appendix:

Table A1. Composition of the ANRS 12249 TasP Study Group (as of January 2016).

Name	Role	Affiliation
Investigators		
François Dabis	Co-PI (France)	• Univ. Bordeaux, ISPED, Centre Inserm U1219 Bordeaux Population Health, Bordeaux, France<br>• INSERM, ISPED, Centre Inserm U1219 Bordeaux Population Health, Bordeaux, France
Deenan Pillay	Co-PI (South Africa)	• Africa Centre for Population Health, University of KwaZulu-Natal, South Africa<br>• Faculty of Medical Sciences, University College London, United Kingdom (UK)
Marie-Louise Newell	Co-PI (United Kingdom)	• Africa Centre for Population Health University of KwaZulu-Natal, South Africa<br>• Faculty of Medicine, University of Southampton, UK
Coordinators		
Collins Iwuji	Trial Coordinator and HIV Clinician (South Africa)	• Africa Centre for Population Health, University of KwaZulu-Natal, South Africa<br>• Research Department of Infection and Population Health, University College London, UK
Joanna Orne-Gliemann	Trial Coordinator (France)	• Univ. Bordeaux, ISPED, Centre Inserm U1219 Bordeaux Population Health, Bordeaux, France<br>• INSERM, ISPED, Centre Inserm U1219 Bordeaux Population Health, Bordeaux, France
Study team		
Till Bärnighausen	Health Economics	• Africa Centre for Population Health, University of KwaZulu-Natal, South Africa<br>• Dept of Global Health & Population, Harvard School of Public Health, Harvard Univ. Boston, USA
Eric Balestre	Epidemiology and Biostatistics	• Univ. Bordeaux, ISPED, Centre Inserm U1219 Bordeaux Population Health, Bordeaux, France<br>• INSERM, ISPED, Centre Inserm U1219 Bordeaux Population Health, Bordeaux, France
Sylvie Boyer	Health Economics	• INSERM, UMR912 (SESSTIM), Marseille, France<br>• Aix Marseille Université, UMR_S912, IRD, Marseille, France<br>• ORS PACA, Observatoire Régional de la Santé Provence-Alpes-Côte d'Azur, Marseille, France
Alexandra Calmy	Adult Medicine	• Service des maladies infectieuses, Hôpital Universitaire de Geneve, Genève, Switzerland
Vincent Calvez	Virology	• Department of virology, Hôpital Pitié-Salpétrière, Paris, France

(*Continued*)

Table A1. Continued.

Name	Role	Affiliation
Rosemary Dray-Spira	Social Sciences	• INSERM U1018, CESP, Epidemiology of Occupational and Social Determinants of Health, Villejuif, France<br>• University of Versailles Saint-Quentin, UMRS 1018, Villejuif, France
Kobus Herbst	Data Management	• Africa Centre for Population Health, University of KwaZulu-Natal, South Africa
John Imrie	Social Sciences	• Africa Centre for Population Health, University of KwaZulu-Natal, South Africa<br>• Centre for Sexual Health and HIV Research, Research Department of Infection and Population, Faculty of Population Health Sciences, University College London, London, UK
Joseph Larmarange	Social Sciences	• Centre Population & Développement (Ceped UMR 196 UPD IRD), Institut de Recherche pour le Développement, Paris, France<br>• Africa Centre for Population Health, University of KwaZulu-Natal, South Africa
France Lert	Social Sciences	• INSERM U1018, CESP, Epidemiology of Occupational and Social Determinants of Health, Villejuif, France<br>• University of Versailles Saint-Quentin, UMRS 1018, Villejuif, France
Thembisa Makowa	Field Operations	• Africa Centre for Population Health, University of KwaZulu-Natal, South Africa
Anne-Geneviève Marcelin	Virology	• Department of virology, Hôpital Pitié-Salpétrière, Paris, France
Nuala McGrath	Epidemiology/Social Sciences	• Faculty of Medicine and Faculty of Human, Social and Mathematical Sciences, University of Southampton, UK<br>• Africa Centre for Population Health, University of KwaZulu-Natal, South Africa<br>• Research Department of Infection and Population Health, University College London, UK
Nonhlanhla Okesola	Nurse Manager	• Africa Centre for Population Health, University of KwaZulu-Natal, South Africa

(*Continued*)

Table A1. Continued.

Name	Role	Affiliation
Tulio de Oliveira	Bioinformatics	• Africa Centre for Population Health, University of KwaZulu-Natal, South Africa
Melanie Plazy	Epidemiology/Social Sciences	• Univ. Bordeaux, ISPED, Centre Inserm U1219 Bordeaux Population Health, Bordeaux, France<br>• INSERM, ISPED, Centre Inserm U1219 Bordeaux Population Health, Bordeaux, France
Camelia Protopopescu	Statistics/Economist	• INSERM, UMR912 (SESSTIM), Marseille, France<br>• Aix Marseille Université, UMR_S912, IRD, Marseille, France<br>• ORS PACA, Observatoire Régional de la Santé Provence-Alpes-Côte d'Azur, Marseille, France
Luis Sagaon-Teyssier	Health Economics	• INSERM, UMR912 (SESSTIM), Marseille, France<br>• Aix Marseille Université, UMR_S912, IRD, Marseille, France<br>• ORS PACA, Observatoire Régional de la Santé Provence-Alpes-Côte d'Azur, Marseille, France
Bruno Spire	Health Economics	• INSERM, UMR912 (SESSTIM), 13006, Marseille, France<br>• Aix Marseille Université, UMR_S912, IRD, Marseille, France<br>• ORS PACA, Observatoire Régional de la Santé Provence-Alpes-Côte d'Azur, Marseille, France
Frank Tanser	Epidemiology and Biostatistics	• Africa Centre for Population Health, University of KwaZulu-Natal, South Africa
Rodolphe Thiébaut	Epidemiology and Biostatistics	• Univ. Bordeaux, ISPED, Centre Inserm U1219 Bordeaux Population Health, Bordeaux, France<br>• INSERM, ISPED, Centre Inserm U1219 Bordeaux Population Health, Bordeaux, France
Thembelile Zuma	Psychology/Social Sciences	• Africa Centre for Population Health, University of KwaZulu-Natal, South Africa

Scientific advisory board

- Chair: Bernard Hirschel (Switzerland)
- International experts: Xavier Anglaret (Ivory Coast), Hoosen Coovadia (South Africa), Alpha Diallo

(France), Bruno Giraudeau (France), Jean-Michel Molina (France), Lynn Morris (South Africa), François Venter (South Africa), Sibongile Zungu (South Africa)
- Community representatives: Eric Fleutelot (France), Eric Goemaere (South Africa), Calice Talom (Cameroon)
- Sponsor representatives (ANRS): Brigitte Bazin, Claire Rekacewicz
- Pharmaceutical company representatives: Golriz Pahlavan-Grumel (MSD), Alice Jacob (Gilead)

Data safety and monitoring board

- Chair: Patrick Yeni (France)
- Members: Sinead Delany-Moretlwe (South Africa), Nathan Ford (South Africa), Catherine Hankins (Netherlands), Helen Weiss (UK)
